# Emergency Transcatheter Aortic Valve Replacement in Cardiogenic Shock Caused by Severe Bicuspid Aortic Valve Stenosis

**DOI:** 10.1016/j.jaccas.2026.109110

**Published:** 2026-07-14

**Authors:** Martin Jurisic, Fabian Starnecker, Michael Joner, Tobias Rheude

**Affiliations:** Department of Cardiovascular Diseases, German Heart Center Munich, Technical University Munich University Hospital, Munich, Germany

**Keywords:** aortic stenosis, bicuspid aortic valve, cardiogenic shock, lifetime management, TAVR

## Abstract

**Background:**

Treatment of severe aortic stenosis in cardiogenic shock remains challenging, particularly in younger patients with bicuspid valve stenosis (BVS).

**Case Summary:**

A 51-year-old woman with severe high-gradient BVS presented in cardiogenic shock with multiorgan dysfunction. Given hemodynamic instability, the heart team proceeded with urgent transcatheter aortic valve replacement. A 26-mm balloon-expandable Edwards SAPIEN 3 Ultra RESILIA valve was successfully implanted, resulting in rapid hemodynamic stabilization.

**Discussion:**

This case highlights the importance of urgent heart team decision-making in unstable patients with severe BVS. Lifetime management requires individualized device selection considering annular dimensions, calcification pattern, coronary access, and future redo-procedure strategies.

**Take-Home Messages:**

In the presence of refractory cardiogenic shock, transcatheter aortic valve replacement may be the preferred treatment strategy and should be considered as part of an individualized lifetime management strategy.

## Graphical Abstract

**Figure:**
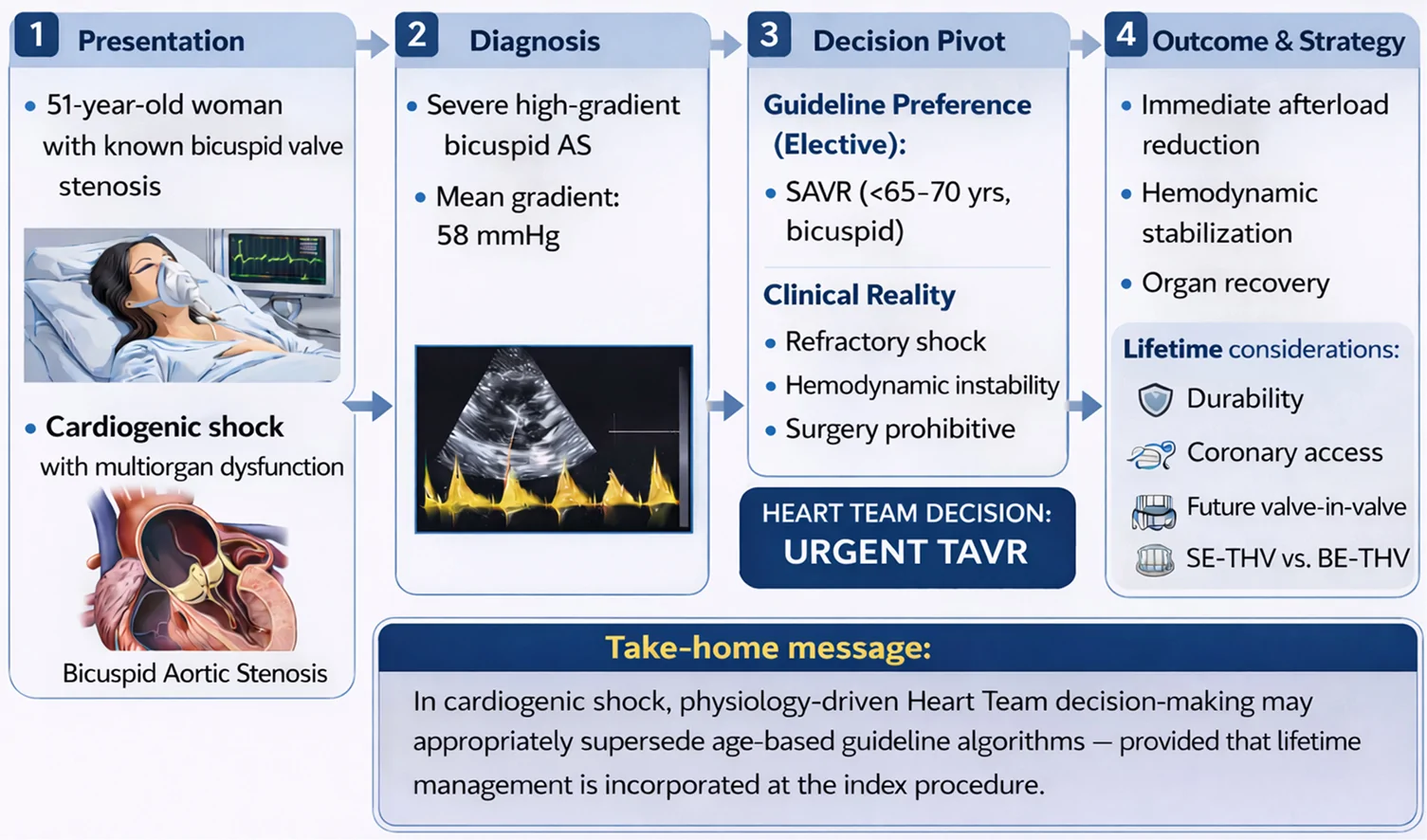

## History of Presentation

A 51-year-old woman presented to the emergency department with worsening dyspnea for 10 days and dizziness.

**Visual Summary:**
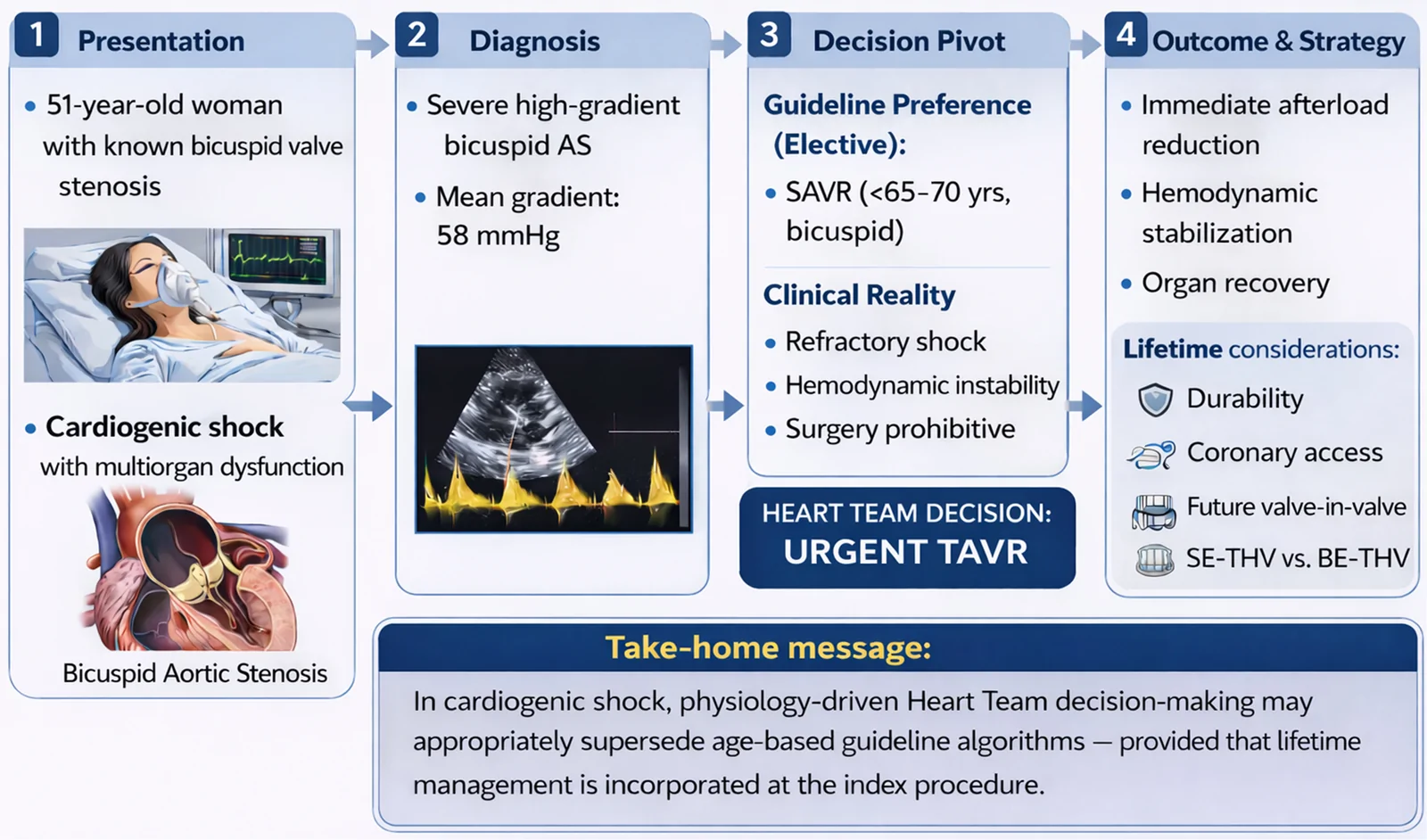
Rescue TAVR in Cardiogenic Shock AS = aortic stenosis; BE-THV = balloon-expandable transcatheter heart valves; SAVR = surgical aortic valve replacement; SE-THV = self-expanding transcatheter heart valve.

## Past Medical History

The patient had a known severe bicuspid valve stenosis for 5 years. Apart from a prior appendectomy, there were no further relevant comorbidities. Cardiovascular risk factors included former smoking (20 pack-years).

## Differential Diagnosis

In this patient presenting with shock and multiorgan dysfunction, the differential diagnosis included acute coronary syndrome, acute decompensated heart failure due to severe aortic stenosis (AS), fulminant myocarditis, stress-induced cardiomyopathy, acute pulmonary embolism, and septic shock.

## Investigations

**Figure 1:**
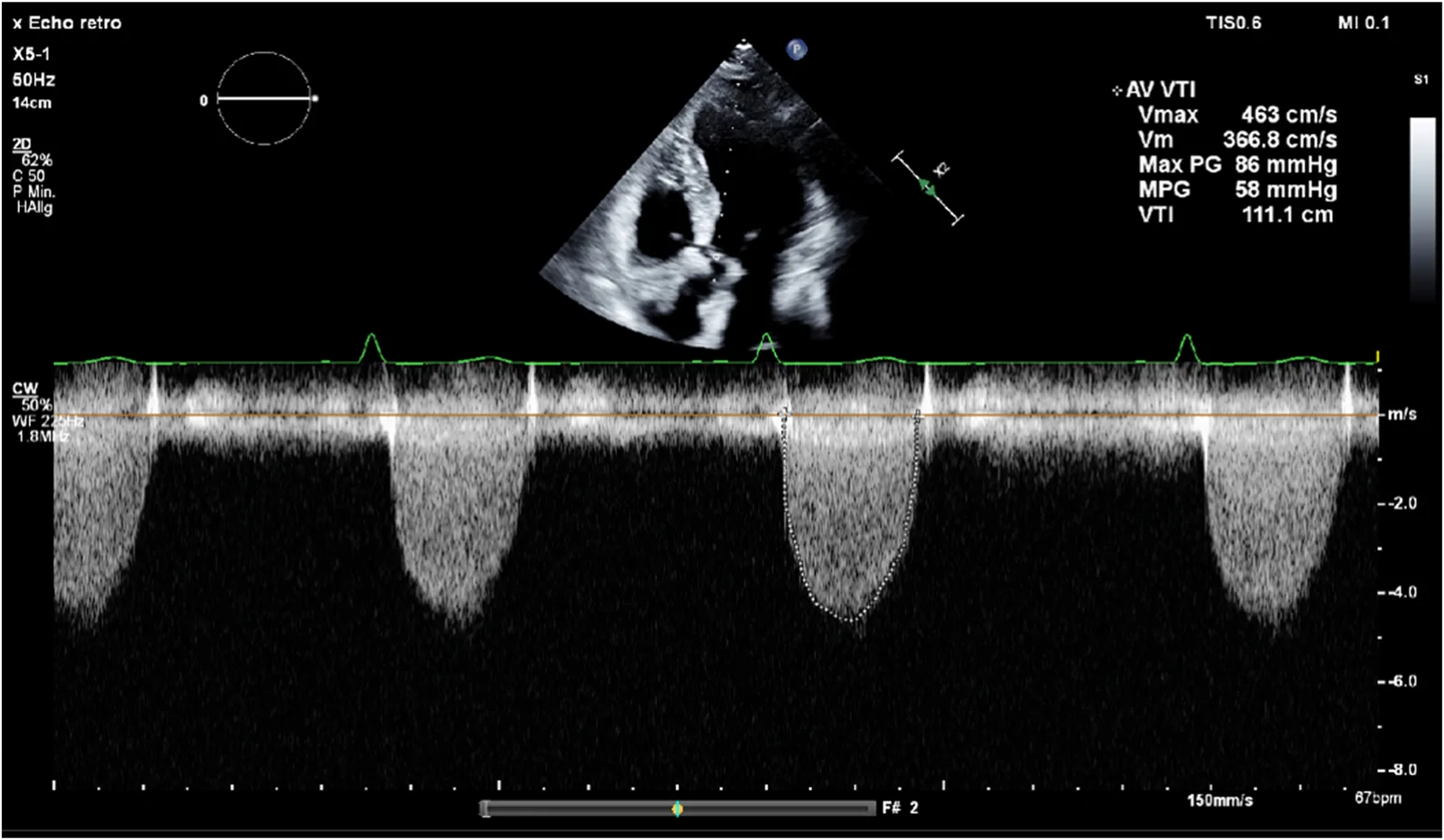
Continuous-Wave Doppler Assessment of Severe Bicuspid Aortic Stenosis Transthoracic echocardiography in an apical 5-chamber view with focused visualization of the left ventricular outflow tract and aortic valve (AV). Continuous-wave Doppler demonstrates markedly elevated systolic velocities consistent with severe aortic stenosis (peak velocity 4.63 m/s; peak gradient 86 mm Hg; mean gradient 58 mm Hg). MPG = mean pressure gradient; VTI = velocity time integral.

On initial presentation, the patient exhibited tachycardia (heart rate 109 beats/min) with borderline hypotension (blood pressure 107/80 mm Hg). During the subsequent clinical course, the patient developed progressive hemodynamic instability with worsening hypotension (82/59 mm Hg). Laboratory testing on admission revealed markedly elevated cardiac biomarkers, increased liver enzymes, rising serum creatinine values, and altered coagulation parameters, reflecting multiorgan dysfunction. Transthoracic echocardiography demonstrated severe, high-gradient bicuspid AS with a mean transvalvular gradient of 58 mm Hg and an aortic valve area of 0.57 cm^2^ with preserved left ventricular function (Figure 1, Videos 1 and 2). Preprocedural computed tomography confirmed Sievers type 0 bicuspid anatomy with excessive calcification (calcium score of 7,374 Agatston units) without relevant left ventricular outflow tract calcification and the following annular dimensions (mean diameter 27.1 mm, area 573 mm^2^, and perimeter 88.2 mm) and a favorable transfemoral access without relevant calcification or stenosis (Video 3).

## Management (Medical/Interventions)

Operative risk stratification revealed a Society of Thoracic Surgeons Predicted Risk of Mortality score of 18.1%. However, it likely underestimates the true operative risk, as it does not fully capture acute cardiogenic shock with evolving multiorgan failure. Given the patient’s hemodynamic instability, coagulopathy, and end-organ dysfunction, the heart team considered the surgical risk to be prohibitive in this acute setting, prompting the decision to proceed with emergent transfemoral transcatheter aortic valve replacement (TAVR). Annular dimensions fueled the discussion to implant either a 26- or a 29-mm Edwards SAPIEN transcatheter heart valve. However, given the extensive valvular calcification and bicuspid anatomy, a 26-mm SAPIEN 3 Ultra RESILIA valve was selected with planned balloon overfilling (+2 mL), following an intentional undersizing strategy to mitigate the risk of annular rupture (Videos 4 to 7).

## Outcome and Follow-Up

The procedure resulted in immediate hemodynamic improvement within minutes after successful valve implantation. The patient was monitored in the intensive care unit and organ function recovered promptly thereafter. Creatinine, creatine kinase-myocardial band, liver enzymes improved markedly within 2 days. The patient was discharged on day 4 after the procedure. At 3-month follow-up, there was sustained valve function, with no relevant valvular or paravalvular regurgitation.

## Discussion

Current international guidelines for valvular heart disease recommend TAVR in patients older than 65 (American College of Cardiology/American Heart Association guidelines) or 70 years (European Society of Cardiology/European Association for Cardio-Thoracic Surgery guidelines) in patients with tricuspid aortic valve stenosis, provided the anatomy is suitable. These recommendations are based on favorable results from large, randomized trials comparing TAVR vs surgical aortic valve replacement (SAVR).^1^, ^2^, ^3^

For stenotic bicuspid valve stenosis, SAVR remains the primary mode of treatment, particularly if these patients are young or have coexistent aortopathy. With the latest European guidelines, TAVR may be considered for the treatment of severe bicuspid aortic valve (BAV) in patients at increased surgical risk, if the anatomy is suitable. Current recommendations are largely driven by the lack of data and the under-representation of BAV in pivotal randomized TAVR trials. Large registries, such as the Society of Thoracic Surgeons/American College of Cardiology Transcatheter Valve Therapy registry and the PARTNER 3 Bicuspid registry, demonstrate a similar mortality with no differences between patients with bicuspid and tricuspid aortic valve, but higher stroke rates in patients with bicuspid valve.^4^^,^^5^ It needs to be underscored that there is huge variability in patients with BAV with regard to the individual anatomy of the aortic valve apparatus; therefore, current guidelines highlight the central role of the multidisciplinary heart team in an individualized decision-making, integrating anatomical feasibility, surgical risk, comorbidities, and overall clinical status.^6^^,^^7^

Valve sizing in bicuspid anatomies remains challenging due to an asymmetric calcium distribution and noncircular annular geometry. The decision to implant a 26-mm valve despite this borderline situation reflects a strategy consistent with contemporary undersizing approaches. Recent data have highlighted the safety and feasibility of intentional annular undersizing in heavily calcified anatomies to reduce the risk of annular injury.^8^ Similarly, dedicated sizing frameworks such as the Annulus, Below Annulus, Commissures, and Leaflet Calcification algorithm emphasize integrating calcium distribution and leaflet morphology rather than relying solely on annular dimensions.^9^ In this patient, excessive calcification and bicuspid morphology favored a more conservative sizing approach combined with controlled balloon overexpansion, balancing the competing risks of annular rupture and paravalvular regurgitation.

Cardiogenic shock in young patients with bicuspid AS is not specifically mentioned in current algorithmic pathways. In contrast to elective cases, where SAVR represents the primary treatment mode for most patients, emergent intervention in the context of refractory cardiogenic shock represents a fundamentally different clinical scenario. This case exemplifies an urgency-driven deviation from age-based recommendations. In the presence of cardiogenic shock and evolving multiorgan dysfunction, including coagulopathy and hemodynamic instability, surgical risk may become prohibitive despite a young chronological age. Under such circumstances, TAVR can serve as a lifesaving rescue therapy by providing immediate afterload reduction and restoration of end-organ perfusion with quick recovery.

Performing TAVR in the setting of cardiogenic shock might require procedural adaptations. Hemodynamic stabilization before valve deployment is critical and may include the use of vasopressors and inotropes. In selected cases, mechanical circulatory support including the use of venoarterial extracorporeal membrane oxygenation needs to be considered. Especially as rapid ventricular pacing is mandatory for predilatation and valve implantation of balloon-expandable valves, which might further contribute to hemodynamic instability. Additionally, the anesthesia strategy plays a crucial role, whereas a minimalist approach, with local anesthesia or conscious sedation, is preferred in elective TAVR, general anesthesia may need to be considered. In the present case, careful pharmacologic hemodynamic management allowed successful valve implantation without the need for mechanical circulatory support or general anesthesia, resulting in immediate afterload reduction and rapid clinical recovery.

Although lifetime management strategies are crucial in younger patients with longer life expectancy, the acute procedural outcome with rapid clinical stabilization needs to be prioritized. Prosthesis selection should take long-term durability, coronary reaccess, feasibility of future redo procedures, and potential surgical explant complexity into consideration. In this regard, TAVR using intra-annular platforms carries the lowest risk of future sinus sequestration when a patient undergoes redo procedure and allows accessibility of the coronaries.^10^

It should also be noted that isolated balloon aortic valvuloplasty may represent a viable treatment option in this context; however, it carries a relevant risk of significant valvular insufficiency. Therefore, whenever feasible, preprocedural computed tomography imaging should be obtained to enable planning for a potential bailout TAVR if necessary.

## Conclusions

Emergent TAVR can be a lifesaving option in unstable young patients with bicuspid AS when surgical mortality is prohibitive, provided a long-term strategy is integrated into procedural planning.

## Funding Support and Author Disclosures

The authors have reported that they have no relationships relevant to the contents of this paper to disclose.

**Take-Home Messages**

• In the presence of refractory cardiogenic shock and multiorgan dysfunction, transcatheter aortic valve replacement is justified as a lifesaving rescue therapy when the surgical risk becomes prohibitive, even in young patients with bicuspid valve stenosis.
• Even in emergent scenarios involving young patients, prosthesis selection should incorporate lifetime management considerations, including valve durability, coronary reaccess, and the feasibility of future valve-in-valve procedures.
• Emergent transcatheter aortic valve replacement in cardiogenic shock requires not only rapid decision-making but also tailored procedural strategies, including valve sizing adaptations and meticulous hemodynamic management.

## Appendix

Video 1 Parasternal Long-Axis View Transthoracic echocardiography demonstrating a calcified bicuspid aortic valve with restricted leaflet motion.

Video 2 Apical 5-Chamber View With Focus on the Aortic Valve Transthoracic echocardiography demonstrating continuous-wave Doppler alignment across the aortic valve.

Video 3 Preprocedural Computed Tomography Assessment Multidetector computed tomography demonstrating type 0 bicuspid anatomy with heavy valvular calcification and suitable transfemoral access.

Video 4 Balloon Aortic Valvuloplasty Predilatation of the native aortic valve using a 25-mm balloon.

Video 5 Valve Deployment Transcatheter implantation of a 26-mm balloon-expandable valve (SAPIEN Ultra 3 RESILIA, Edwards Lifesciences).

Video 6 Postdilatation Postdilatation using the delivery balloon with an additional 2 mL volume.

Video 7 Final Angiographic Result Final angiography demonstrating an appropriate valve position, preserved coronary flow, and no significant aortic regurgitation.
